# Tamponade dressings versus no tamponade after hemorrhoidectomy: study protocol for a randomized controlled trial

**DOI:** 10.1186/s13063-019-3280-0

**Published:** 2019-04-02

**Authors:** Mike Ralf Langenbach, Dörthe Seidel

**Affiliations:** 1 Klinik für Allgemein-/Viszeralchirurgie und Koloproktologie, Helios St. Elisabeth Klinik, Josefstr. 3, 46045 Oberhausen, Germany; 2 Institut für Forschung in der Operativen Medizin (IFOM), Universität Witten/Herdecke, Ostmerheimerstraße 200 Haus 38, 51109 Köln, Germany

**Keywords:** Hemorrhoids, Hemorrhoidectomy, Postoperative pain, Postoperative bleeding, Tamponade, Anal tampon

## Abstract

**Background:**

Symptomatic hemorrhoids are one of the most common anorectal disorders. Many surgeons use tamponades after open hemorrhoidectomy to manage postoperative bleeding. The question of whether a tamponade is necessary and beneficial after hemorrhoidectomy has not yet been conclusively answered. A previously conducted single-center pilot trial included 100 patients after Milligan–Morgan hemorrhoidectomy. The data indicated that insertion of an anal tamponade after hemorrhoidectomy does not reduce postoperative bleeding but causes significantly more pain. The findings of this pilot trial are now to be verified by means of a multicenter randomized clinical study called NoTamp.

**Methods:**

We plan to include 953 patients after Milligan-Morgan or Parks hemorrhoidectomy in the NoTamp study. The aim is to demonstrate that using no tamponade dressing after open hemorrhoidectomy is not inferior to using tamponades with respect to postoperative bleeding, and that the patients report less pain. Primary endpoints of the trial are the maximum postoperative pain within 48 h and the incidence of severe postoperative bleeding that requires surgical revision within 7 days after the surgical procedure. Secondary endpoints of the study are the use of analgesics in the postoperative course, the lowest hemoglobin documented within 7 days, quality of life and patient satisfaction. Safety analysis includes all adverse and serious adverse events in relation to the study treatment. Further information can be found in the registration at the German Registry of Clinical Studies (DRKS00011590) and on the study webpage (https://notamp.de/en-GB/trial/main/setLocale/en_GB/). The study is financed by the HELIOS research funding.

**Discussion:**

The study received full ethics committee approval. The first patient was enrolled on 3 May 2017. This trial will finally answer the question whether the insertion of a tamponade after open hemorrhoidectomy is necessary and beneficial.

**Trial registration:**

German Clinical Trials Register (Deutsches Register Klinischer Studien (DRKS), DRKS00011590. Registered on 12 April 2017.

## Background

Symptomatic hemorrhoids are one of the most common anorectal disorders [1–3]. The exact incidence of hemorrhoid disease is difficult to quantify and published epidemiologic data are rather old and rare. The prevalence is approximately 4% in the United States of America, [4] with presentation peaking between the ages of 45 and 65 years. Since the second half of the twentieth century, there seems to have been an unexplained decrease in the prevalence of symptomatic hemorrhoid disease in both the USA and England [5]. It is estimated that approximately 50% of all Germans develop symptomatic hemorrhoids during their lifetime and some authors even suggest that up to 70% of the adult population is affected by hemorrhoids [6]. However, these numbers are not based on epidemiological data, but are estimated by experts [7]. Symptomatic hemorrhoids are very common in both men and women. About half of all people aged 50 years have symptomatic hemorrhoid disease. About 40% of individuals with hemorrhoids are asymptomatic [1].

Symptomatic hemorrhoid disease has a large impact on quality of life, and can be managed with a multitude of surgical and nonsurgical treatments [8]. Treatment options comprise conservative and surgical therapy applied according to individual patient and clinical factors [9]. Continued symptoms despite conservative or minimally invasive measures usually require surgical intervention, and surgery is the initial treatment of choice in patients with symptomatic grade IV hemorrhoids or those with strangulated internal hemorrhoids [8]. It may also be required for symptomatic grade III hemorrhoids and in patients who present with thrombosed hemorrhoids.

In recent years a variety of surgical procedures has become available for treating symptomatic hemorrhoids, but high (Parks) or low (Milligan-Morgan) ligation with excision is still one of the common performed surgeries. The most commonly used method for treating third-degree and fourth-degree enlarged hemorrhoids is still Milligan-Morgan’s surgical procedure [2, 3, 10–13]. The open hemorrhoidectomy is often the preferred approach to surgically treat severe acute gangrenous hemorrhoids where tissue edema and necrosis preclude closure of the mucosa [14].

Hemorrhoidectomy is generally performed as an inpatient procedure [12], as it may be associated with significant postoperative complications like pain, bleeding, pruritus and mucus production. The occurrence of bleeding is often associated with the first passage of hard stool after surgery. Early bleeding can also result from inadequate thrombus formation. Postoperative bleeding can be a serious complication that may range from causing discomfort to being potentially life-threatening. Postoperative bleeding may occur in 2–6% of the cases [15–17], with early bleeding being more common than late bleeding [18, 19].

Depending on the extent of dissection and due to the presence of incisions below the dentate line, postoperative pain can be severe, and may delay return to normal activities for several weeks. Pain can usually be managed with oral analgesics, avoidance of constipation, and sitting in a bath.

Postoperative bleeding may occur in 1–2% of patients 1 week after surgery as a result of eschar separation and is usually self-limited [20]. Patients requiring a combination of aspirin and thienopyridine derivatives (e.g., clopidogrel) have a heightened risk of bleeding [21].

In order to avoid postoperative bleeding as well as pain after hemorrhoidectomy, surgical techniques and instruments have been improved and further developed [22]. Additionally, researchers investigated stool softeners and oral administration of metronidazole [12].

In order to control postsurgical bleeding, many surgeons insert an anal tampon, but little scientific attention has been paid to the necessity of local dressings after hemorrhoidectomy. Benefit and possible disadvantages (e.g. pain) of the tamponades are under discussion.

Two randomized studies evaluated the effects of anal tampons after hemorrhoidectomy. Myles et al. compared a simple, non-adherent wound pad with a gelatin sponge plug [23]. Ingram et al. performed a study comparing a calcium alginate dressing with standard gauze packing after hemorrhoidectomy [24]. Both studies focused on the effects of different types of tamponades on the severity of pain and bleeding. Nevertheless, the fundamental question of whether tamponade is really necessary and beneficial after hemorrhoidectomy has never been answered in a comparative study.

Within a previously conducted single-center randomized controlled pilot study performed by the same study group (DRKS-ID: DRKS00004568) 100 patients surgically treated with Milligan-Morgan hemorrhoidectomy have been randomized to be treated either with a postoperative anal tampon (48 patients) or without (52 patients) [25]. The primary endpoint of the study, maximum pain intensity (0–10), has been shown to be significantly lower in patients without tamponade (4.2) than in the group with tamponade dressing after surgery [1, 6] (*p* = 0.001). Complications occurred in 7 patients (15%) in the group with tamponade and in 11 patients in the group without tamponade (21%) (*p* = 0.444), whereas the number of patients with severe anal bleeding was small in both groups (*n* = 2 with tamponade and *n* = 5 without tamponade). Bandage changes were required less often in the group treated without tamponade (*p* = 0.013). The average length of hospital stay was 4 days in both groups.

The results of the pilot study indicate that using tamponade after hemorrhoidectomy significantly increases postoperative pain but has no value in avoiding postoperative bleeding. With the exception of patients on anticoagulants, who have a heightened risk of severe postoperative bleeding, the use of anal tamponade dressings after hemorrhoidectomy seems not to be necessary in routine care.

The results of the pilot study should now be verified by means of a multicenter randomized clinical trial (RCT). This multicenter RCT aims to prove that the established postoperative routine of using tamponade after hemorrhoidectomy has no additional benefit in avoiding postoperative bleeding but leads to significantly more postoperative pain. The NoTamp-study aims to create sufficient evidence base for decision-making on the necessity of using tamponade dressings after hemorrhoidectomy.

## Methods/design

### Aim of the study

The primary aim of the study is to prove that in routine clinical practice, treating patients without a tamponade dressing after hemorrhoidectomy results in a lower pain burden, and is non-inferior to the use of tamponade in terms of the incidence of severe postoperative bleeding requiring surgical revision. 

Secondary aims are to assess all patients in the group without primary tamponade dressing after surgery who needed emergency tamponade, and to compare differences between the study groups in preoperative and postoperative hemoglobin values and the use of pain medications. Quality of life and patient satisfaction will also be compared between the two treatment groups.

The safety analysis aims to compare the number of adverse events (AE) and serious adverse events (SAE) in the two study groups in order to make a statement on the potential risk to patients without a tamponade dressing after hemorrhoidectomy.

### Design

The NoTamp study is designed as a German national, multicenter, randomized controlled clinical non-inferiority trial. Participants are randomly allocated in a 1:1 ratio to each treatment group. Randomization will be stratified per study site. Allocation to treatment groups will be performed using a centralized web-based tool. Randomization is recommended to be performed at the earliest 12 h before the start of surgery. The patient will not be informed about the result of randomization before surgery. The duration of the clinical trial for each randomized patient is 7 days. There will be no blinding of participants, physicians, nurses or outcome assessors. No interim analysis is planned.

### Setting

This multicenter study will be conducted in hospital departments with a special focus on colonic and rectal surgery. Participating study sites are general and visceral surgery departments with at least one expert who has passed the German examination for specialization in proctology. The study is mainly performed within but not limited to the Helios clinic group. The majority of the participating study sites are hospitals for basic and standard care. Two study sites are maximum care hospitals. The Helios clinic group ensures uniform quality management and standardized documentation in the clinical file. For trial sites outside Helios, suitability for participation will be assessed before inclusion in the study. Study sites are located all over Germany. The number of study sites may be adjusted according to the frequency of patient recruitment.

In order to ensure the best possible transferability of the study results into the clinical routine, local clinical standards are applied, evaluated with regard to their influence on the results, and fully reported. Patients are provided with the best clinical standard.

### Participants

The target population of this clinical study includes patients with symptomatic hemorrhoids, who require wound treatment after Milligan-Morgan or Parks hemorrhoidectomy.

All adult patients (age ≥ 18 years) suffering from symptomatic hemorrhoids of grade III or IV and scheduled for hemorrhoidectomy by the Milligan-Morgan or Parks procedure are eligible for the NoTamp study. Study participants must be fully legally competent and need to provide written informed consent before randomization, inclusion in the trial, and any trial-related procedure.

Patients with inflammatory anal diseases such as abscesses, fistulas, or gangrene, and pregnant women (according to the patient’s self-report) will be excluded from participation in this clinical study. Patients who are in a dependency and/or employment relationship with the study leader or any of the study staff are not allowed to be included in the trial.

Any inability of the patient to respond to requests, to adequately assess risk, or to comply with the requirements of the course of study, both in the inpatient and outpatient therapy episode, must be considered as non-compliance. If, in the estimation of the clinical investigator, a patient is non-compliant at the time of inclusion or is expected to be non-compliant during the course of the trial, this patient may not be included. Patients participating in other clinical trials that are expected to affect the outcome of the NoTamp-study will not be included.

**Fig. 1:**
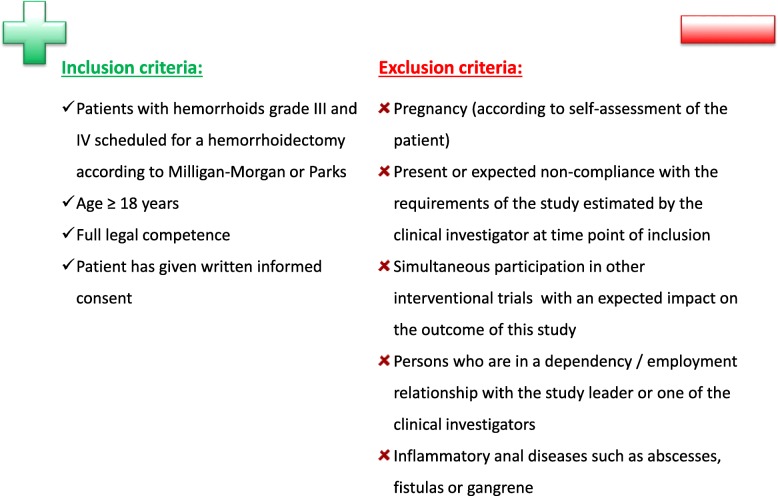
Inclusion and exclusion criteria

Figure 1 provides a full overview of the inclusion and exclusion criteria. Reasons for non-enrolment or non-eligibility will be documented for all patients who fulfil the inclusion criteria but who are not included in the trial.

### Intervention and comparison

In the intervention group, no tamponade dressing will be placed in the rectum of the patient after achieving complete hemostasis. The rectum and anus will be cleaned and a pad with high-absorbence capacity will be placed on the aperture to absorb potential blood loss. The pad will be fixed with mesh pants.

In the control group a tamponade dressing is to be placed in the patient’s anal canal and lower rectum after complete hemostasis. The intraoperatively inserted tamponade remains in the rectum of the patient at least until first defecation and possible spontaneous removal, but usually for a maximum of 24 h. Tamponade dressings may be used according to clinical routine. The type of tamponade used for the study participants will be recorded in the case report forms (CRF).

### Previous and concomitant medication and therapy

The NoTamp study focuses on the evaluation of post-surgical wound treatment. The surgical procedure takes place followed by postoperative care. The surgical procedures allowed in this study include Milligan Morgan’s open hemorrhoidectomy and Park’s submucosal hemorrhoidectomy. Both surgical procedures remove the excessive hemorrhoidal tissue and result in an open wound that needs further treatment.

Thrombosis prophylaxis with low-molecular-weight heparin is mandatory for all patients. Rectal cleansing (preoperative), urinary catheters, analgesics, stool control, and topical applications (ointments) are optional. Discharge management and postoperative controls are usually customized to the individual patient. Pain therapy and prophylaxis include local anesthetics, opioids, non-steroidal anti-inflammatory drugs, muscle relaxants, and other medications such as diosmin and/or sucralfate. During the postoperative period in the recovery room, the patient may receive intravenous or oral pain medication as needed. Subsequently, patients in both treatment groups may receive analgesics as needed until a correspondingly low level of pain (numeric rating scale (NRS) ≤ 3) or freedom from pain is achieved.

After hospital discharge, patients are asked to record their pain medication with type, frequency and amount of intake and to provide this information for documentation in the CRF at the study visit after 7 days. An appropriate supporting documentation template will be provided for the patient.

All previous and concomitant medication will be administered as per clinical routine and according to the patient’s needs and demands. All drugs and other treatments administered will be recorded in the CRF. Since this study represents an evaluation of clinical routine, no treatment guidelines are given.

### Primary and secondary outcomes

Primary outcomes of the NoTamp study are maximum postoperative pain, as measured by a numerical rating scale (NRS) at four time points within 48 h after the surgical procedure, and the occurrence of postoperative bleeding with the need for surgical revision within 7 days after hemorrhoidectomy. Postoperative pain is measured at rest within routine care at 6, 12, 24, and 48 h after the surgical procedure. The most severe pain within 48 h of the surgical procedure will be compared between the treatment groups.

As a secondary outcome, the postoperative pain at each predetermined evaluation time point (6, 12, 24, and 48 h and 7 days after surgery) will be compared between treatment groups. Additionally, the change in pain intensity from 6 h after the surgical procedure to 12, 24, and 48 h and day 7 after the procedure and the development of pain between 24 and 48 h will be compared between the intervention and control group. The number of patients in the group without primary tamponade who require tamponade dressings will also be documented.

Further secondary endpoints that will be compared between the treatment groups are the use of analgesics; the lowest hemoglobin documented in clinical routine within 7 days; quality of life using EuroQuol 5 dimensions (EQ. 5D) at screening, on discharge and on day 7 and patient satisfaction on day 7. Patient satisfaction will be measured using the key figures “subjective treatment errors”; “subjective treatment success”; the satisfaction index and the expectation fulfillment scale of the Cologne Patient Satisfaction Questionnaire of Pfaff et al. [26].

**Fig. 2:**
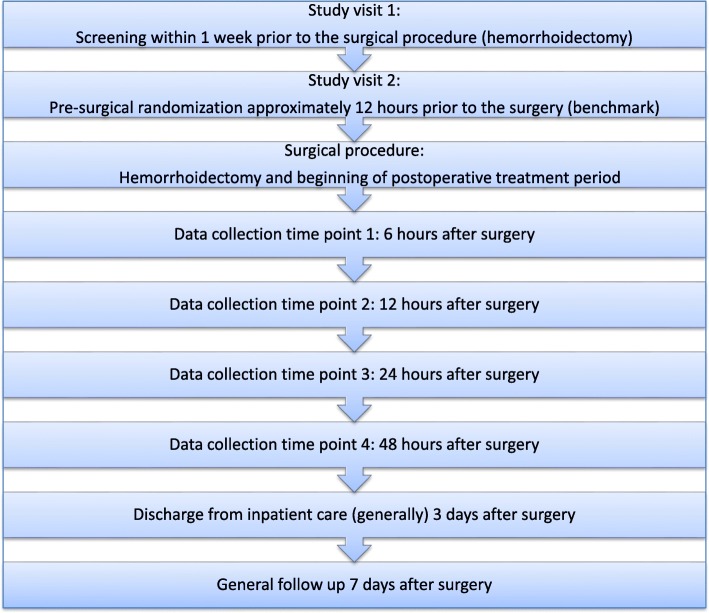
Visit plan. Schematic overview of study visits and postsurgical pain assessment time points

**Fig. 3:**
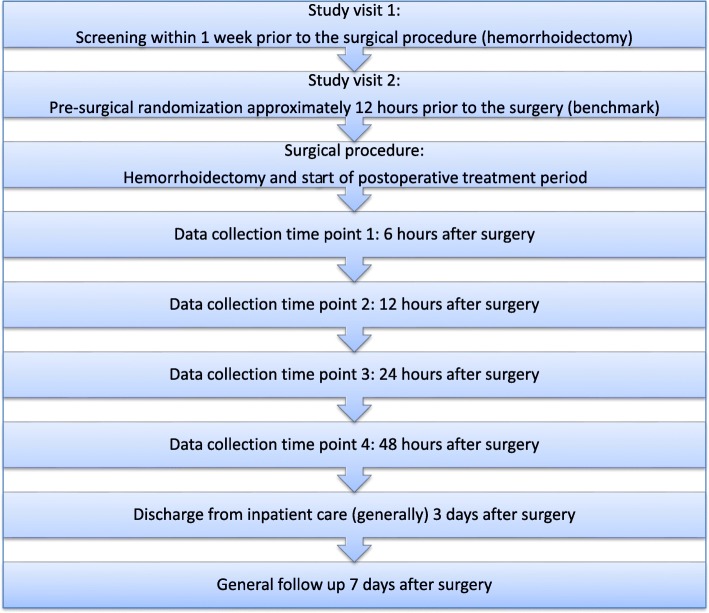
Schedule of enrolment, interventions and assessments according to SPIRIT

For safety analysis all expected and unexpected AEs and SAEs occurring in temporal relation to the clinical trial will be documented and compared between the treatment groups. Documentation includes the causal relationship with the therapy to be investigated and/or with the previous surgical treatment. The nature of the event and the onset, end, and severity (mild, moderate, severe) of each AE will be documented. A full overview of the visit plan and the schedule of enrollment, interventions, and assessments are provided in Figs. 2 and 3.

### Statistical analysis and power calculation

The analysis of the primary and secondary endpoints will be performed according to an adapted intention-to-treat (ITT) principle. There will be no interim analysis. The ITT analysis population will include all randomized participants who have undergone the surgical procedure (hemorrhoidectomy). The ITT analysis population will comprise participants assigned to the treatment group based on the randomization schedule, regardless of the treatment actually received. Randomization will be performed up to 12 h prior to the surgical intervention. However, without the surgical intervention there will be no postoperative wound that requires further treatment and thus there will be no data collected and analysis will not be possible. The study participant will therefore be excluded from the clinical trial if the scheduled surgery is not performed. Patients who are excluded from the trial after randomization and before the start of the observation period (postoperative wound care with or without tamponade) are replaced by another study participant in order to achieve the required number of cases. The randomization sequence will be continued. The next patient does not receive the randomized allocation of the excluded patient. The study will be continued until the required number of evaluable patients has been reached. Due to complications it may not be possible to comply with the randomly assigned treatment regimen in a very small number of patients. These patients will not be excluded from the study. Study participants will also be excluded from the clinical trial if a surgical procedure other than Milligan-Morgan or Parks is performed, which does not result in an open wound.

Details of participants’ baseline characteristics including preoperative pain and hemoglobin levels will be provided, but the data will not be compared statistically (by calculating the *p* value) because any differences between groups at this point must arise by chance (if randomized properly).

As a secondary approach, a per-protocol (PP) analysis will be performed excluding patients with any serious protocol deviations, if applicable. The safety analysis will be performed based on the as-treated (AS) population.

In order to address the primary aim of the study to prove that treating patients without a tamponade dressing after hemorrhoidectomy results in a lower pain burden while the incidence of bleeding requiring surgical revision is no greater than in the patients treated with tamponade (non-inferiority), the primary outcome variables will be examined in terms of an a priori ordered hypothesis at a level of significance of 0.05. Pain values will be described by the mean (SD) and median (IQR). The last observation carried forward (LOCF) method will be used to replace missing values for pain, but only if at least one documentation of postoperative pain is available. Differences in pain will be analyzed using the non-parametric U test (two-sided; alpha = 0.05). If significantly lower pain levels are detected in the treatment arm without tamponade, non-inferiority in the bleeding rate will be also be analyzed at the 5% significance level. Alpha error correction is not required [27]. The incidence of postoperative bleeding needing revision (bleeding rate) will be determined in both treatment arms and will be tested for non-inferiority by calculating the 90% confidence interval (CI) (one-sided hypothesis), with a rate of bleeding two times higher considered acceptable, whereas a bleeding rate three times higher will no longer be considered non-inferior. This rate difference (together with the 90% CI margins) will be considered in relation to the observed revision rate in the control arm. This gives the estimator for the relative increase together with the confidence margins. If the upper limit of this relative increase exceeds 3, then a threefold increase is still possible and non-inferiority is rejected. If the upper limit of this relative increase is < 3, non-inferiority is assumed (with an error rate of 5%). In the unexpected case that the revision rate in the control group is rather low (< 2%) or even 0%, the relative increase will not be calculated. In this case, only the revision rates in the treatment groups are reported (with the 95% CI). The number of patients requiring tamponade in the postoperative course in the group that did not receive primary tamponade after surgery will be presented with the 95% CI.

Pain values at all predetermined survey times and the development of pain between selected time points will be compared in the two treatment groups using the U test. The lowest hemoglobin values in the postoperative period up to day 7 will be compared in the two treatment groups using the two-sided *t* test. The amount and type of postoperative pain medication will be described by percent values for each treatment group. For the safety analysis the incidence of AEs and SAEs by category (severity) will be compared in the two treatment groups using Fisher’s exact test.

There will be no a priori planned subgroup analysis, but a multivariate analysis of all study patients (ITT population) will be performed investigating the occurrence of severe postoperative bleeding (dependent variable). In this logistic regression, the following variables are considered as possible predictors (independent variables): age, gender, surgical method used (Milligan-Morgan or Parks), severity of hemorrhoids (grade III/IV), use of tamponade (yes/no), type of tamponade used, type of anesthesia used, occurrence of intraoperative complications, preoperative hemoglobin levels, type and amount of pain therapy (in particular the performance of a pudendal block), and type and amount of anticoagulation therapy. If this multivariate analysis shows that there is a significant predictor of bleeding that is not evenly distributed within the two study groups (significance in demonstrating the comparability of the two groups), a subgroup analysis of this variable will be performed. In this case, primary and secondary endpoints will be analyzed as described previously, in the subgroups of the unequally distributed variable.

Mental illness in the study participants, preoperative pain levels, the use and type of concomitant pain therapy, and the type of tamponade used represent factors possibly influencing pain, quality of life, and patient satisfaction. The influence of these factors on the aforementioned outcomes will be examined.

Based on the results of the single-center pilot study that demonstrated significant pain reduction (first primary outcome) without tamponade, it can be assumed with great probability that an advantage can also be demonstrated in this much larger multicenter study (first hypothesis). For the second primary outcome based on the results of the pilot study and the international literature it is predicted that 4% of the patients will need surgical revision with tamponade due to severe postoperative bleeding in the treatment group (96% estimated success rate in the control group).

In the intervention group without tamponade a slightly higher rate of bleeding needing surgical revision of ca. 8% is expected. The bleeding rate was 10% in 52 cases included in the pilot study. Therefore, the clinically acceptable and justified limit for non-inferiority is set at 12%, or 8% higher than in the control group with tamponade (8% non-inferiority limit).

Based on these assumptions, the sample size is 433 per group, or 866 patients in total. For study enrollment, assuming a maximum 10% rate of loss to follow up, 953 patients are required. The power was set at 80% in all calculations. Detailed procedures for executing the statistical analysis of the primary and secondary variables and other data will be provided in a statistical analysis plan.

## Discussion

Inserting tamponade after surgery resulting in an open wound in the rectum represents a long-standing and commonly applied clinical standard in Germany. The results of the previously performed single-center randomized controlled pilot trial published in 2014 indicate that insertion of an anal tampon after hemorrhoidectomy does not reduce postoperative bleeding but causes significantly more pain [25]. This multicenter RCT will be conducted to prove that anal tampons should not be used routinely after open hemorrhoidectomy but may be considered when specific indications justify its use.

Since the NoTamp-study represents an evaluation of a treatment method in clinical routine, no guidelines are given on concomitant medication or other concomitant therapeutic measures. A lack of standardization of the concomitant medication may have a negative impact on the results of the study. Thus, all concomitant therapy measures are documented and their influence on the study outcome will be analyzed. The study focuses on high transferability of the results into clinical routine.

Methods to minimize bias were implemented whenever possible. Not blinding study participants and investigators regarding the treatment assingment as well as performing the randomization up to 12 h prior to surgery may be seen as a limitation of this study. Performing randomization with allocation concealment will ensure that patients on anticoagulation therapy or pre-existing pain medication or those with specific oncological colorectal and gynecological diseases are evenly distributed between the experimental and the control group. The same conditions will be created within both treatment groups. Due to the characteristics of the postoperative wound treatment with tamponade, which is clearly evident to the patient, the blinding of study participants cannot be achieved. The blinding of the attending physician or the nurses is also not feasible due to the nature of the treatment process. Blinding of outcome assessors is an important method against bias that unfortunately cannot be implemented in this study. In most of the participating clinics there are no study-supporting facilities. External personnel could not be financed. According to the investigators, blinding of the clinic’s internal staff is not possible due to the clinical routine or because the personnel resources are not available. Prior to any trial-related procedure the investigators will obtain written informed consent from every potential study participant.

The randomization will be performed using a centralized web-based tool on a website created for the NoTamp study [28]. The website provides individualized access for every clinical investigator. The front page informs patients and scientists about the study in German and English and a share point in the protected area ensures unrestricted access to all study documents 24 h per day.

Due to organizational reasons, the randomization process using the web-based tool needs to be performed on the day before the surgical procedure is scheduled. The procedure of intraoperative randomization was discussed in detail with the investigators. However, it could not be implemented.

The study visit plan is adapted to the requirements of the clinical routine whenever possible in order to ensure complete and trouble-free data collection. Pain assessment may be a problem at some time points when the patient may be unavailable to provide a statement e.g. due to sleeping. Missing data are expected in these cases, but they should be to a minimum. During outpatient care, templates will be provided for patient’s self-assessment of pain in order to ensure complete documentation.

As neither the German Medical Devices Act nor the German Medicines Act is applicable to this study, the study does not require a sponsor. The study leader is fully legally responsible and delegates the activities of study planning, conduct of the study, analysis, and reporting to the Institute for Research in Operative Medicine (IFOM) of the Private University of Witten/Herdecke. The division of Clinical Research of the IFOM represents the central organizational unit of the study. All study activities are performed by the project manager and the clinical research associates (CRA). Periodical monitoring to ensure appropriate data quality will be performed by two independent (CRA) at each participating study site. The purpose of monitoring is to assure the rights and safety of all study participants; the validity, verifiability, and completeness of the study data and the conformity of the study with the study protocol, good clinical practice (GCP) and the applicable legal provisions. First monitoring will be performed at the latest after inclusion of the first three patients in order to detect potential problems at an early stage. The overall frequency of monitoring will be adapted depending on the number of patients included and the quality of the data.

An advisory board will oversee the planning, implementation and evaluation of the clinical trial. This includes reviewing the study protocol, obtaining regular information about the progress of the study and any serious adverse events that have occurred, and critically reviewing the final report. The members of the advisory board are free to attend the study meetings, but are not obliged to do so.

## Trial status

This study protocol is published using version 3 of 7 March 2017 as approved by the EC Witten/Herdecke. Recruitment started on 5 May 2017 and is estimated to be completed in June 2021. Current information on the participating study sites and the recruitment status can be found on the study website (https://notamp.de/en-GB/index/login/setLocale/en_GB/). The number of patients included in the study is shown in the box at the bottom left for each study site.

## Abbreviations

AE: Adverse event
AS: As treated
CI: Confidence interval
CRA: Clinical research associates
CRF: Case report forms
EQ 5D: EuroQuol 5 Dimensions
IFOM: Institute for Research in Operative Medicine
IQR: Interquartile range
ITT: Intention to treat
LOCF: Last observation carried forward
NRS: Numerical rating scale
PP: Per protocol
RCT: Randomized controlled trial
SAE: Serious adverse event
SD: Standard deviation
