# Correction to: Tamponade dressings versus no tamponade after hemorrhoidectomy: study protocol for a randomized controlled trial

**DOI:** 10.1186/s13063-019-3358-8

**Published:** 2019-05-20

**Authors:** Mike Ralf Langenbach, Dörthe Seidel

**Affiliations:** 1 Klinik für Allgemein-/Viszeralchirurgie und Koloproktologie, Helios St. Elisabeth Klinik Josefstr. 3, 46045 Oberhausen, Germany; 2 Institut für Forschung in der Operativen Medizin (IFOM), Universität Witten/Herdecke, Ostmerheimerstraße 200 Haus 38, 51109 Köln, Germany

**Correction to: Trials**

**https://doi.org/10.1186/s13063-019-3280-0**

After publication of the original article [1], the authors have notified us that their originally submitted Table 1 was mistakenly replaced with Fig. 3 (duplicate of Fig. 2) during editing. The original article has been corrected.

**Table 1:** Schedule of enrolment, interventions and assessments according to SPIRIT

	Enrolment		Surgery	Post Surgical Pain Assessment				Follow UP 1 Hospital Discharge	Follow up 2 End of Study
Time Points (t)	t -7 days	t-12 hours	t 0	6 hours	12 hours	24 hours	48 hours	Day 3	Day 7
Eligibility Screening and Allocation To Treatment Groups									
Obtaining Written Informed Consent	x								
Checking In- and Exclusion criteria	x								
Web-based Randomisation		x							
Allocation			x						
Interventions									
No Tamponade Dressing			x						
Tamponade Dressing			x						
Assessment of Baseline Variables									
Demography	x								
Goligher`s Classification of Hemorrhoids	x								
Concomitant Medication & Comorbidities	x		x						
Hemoglobin (as part of the clinical routine)	x		x						
Pain Prophylaxis and Treatment			x						
Description of Surgery			x						
Assessment of Outcome Variables									
Change of Concomitant Medication & Comorbidities (including pain medication)								x	x
Pain Prophylaxis and Treatment								x	x
Tamponade Dressing Removal or Insertion			x					x	x
Hemoglobin (as part of the clinical routine)	x		x					x	x
Pain	x			x	x	x	x	x	x
Quality of Life (EQ5D)	x							x	x
Patient Satisfaction									x
Surgical Revision due to Bleeding								x	x
Adverse events & Serious adverse events			x					x	x

Legend: Detailed overview of all study time points with description of data collection during enrolment, postsurgical pain assessment and follow up

The publisher apologizes to the readers for the inconvenience caused.

Table 1, as well as the updated reference can be found below.

- Correct reference in the last sentence of within the Primary and secondary outcomes: “A full overview of the visit plan and the schedule of enrollment, interventions, and assessments are provided in Fig. 2 and Table 1.”

## References

[1] Langenbach, Seidel. Tamponade dressings versus no tamponade after hemorrhoidectomy: study protocol for a randomized controlled trial. 2019;20:188. 10.1186/s13063-019-3280-0. 10.1186/s13063-019-3280-0 PMC6444871 30940201

